# Supplementary figures and images for: Comparison of soil bacterial community and functional characteristics following afforestation in the semi-arid areas
Source: PeerJ. 2019 Jun 25;7:e7141. doi: 10.7717/peerj.7141 (PMC6598672; doi:10.7717/peerj.7141)

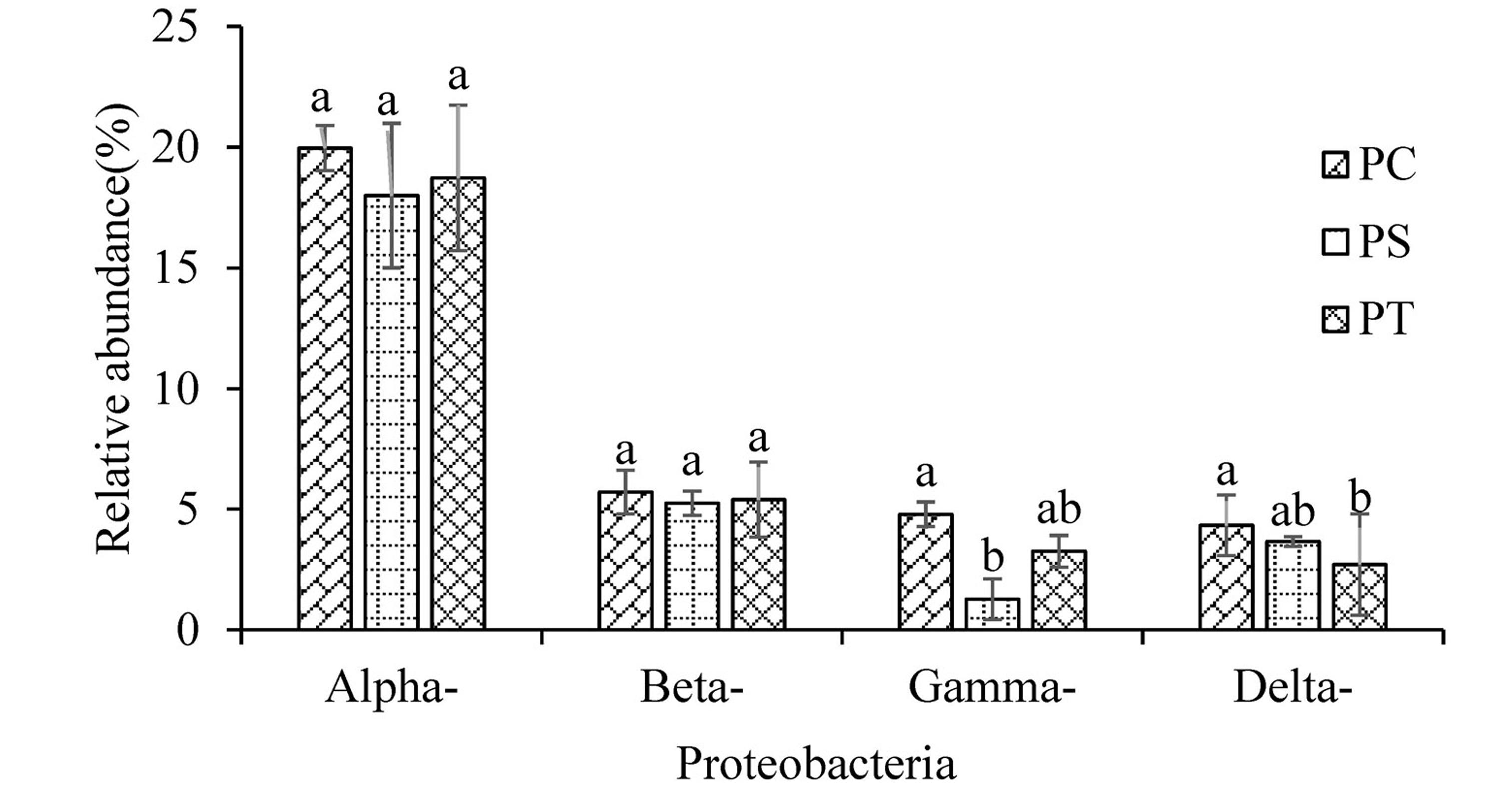

Supplement: Figure S1 — The length of the curve reflects the size of the sample sequencing. The longer the curve, the higher the depth of the sequencing, and the greater the possibility of observing higher diversity. The flatness of the curve reflects the impact of the sequencing depth on the diversity of the observed samples. The flatter the curve indicates that the sequencing result is sufficient to reflect the diversity of the current samples, and the further increase of the sequencing depth is unable to detect a large number of new OTU that have not been discovered. On the contrary, it indicates that the diversity is not close to saturation, and further increasing the sequencing depth will help to observe more new OTU. PC: Populus × canadensis Moench; PS: Pinus sylvestris var. mongolica; PT: Pinus tabuliformis. [file peerj-07-7141-s001.png]

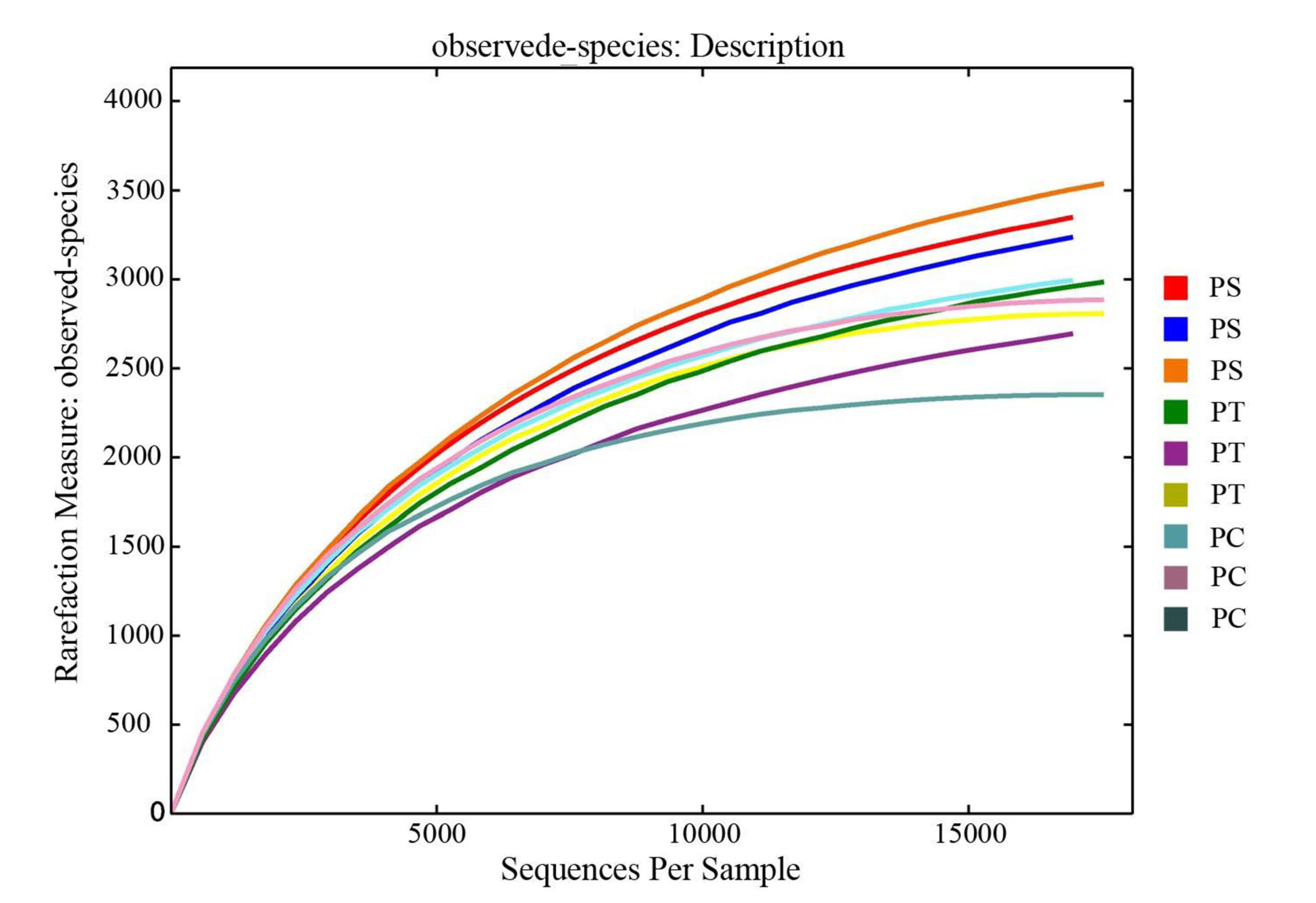

Supplement: Figure S2 — PC: Populus × canadensis Moench; PS: Pinus sylvestris var. mongolica; PT: Pinus tabuliformis. [file peerj-07-7141-s002.png]
