# Supplementary material for: Comparison of soil bacterial community and functional characteristics following afforestation in the semi-arid areas
Source: PeerJ. 2019 Jun 25;7:e7141. doi: 10.7717/peerj.7141 (PMC6598672; doi:10.7717/peerj.7141)
Supplement: Table S1 [file peerj-07-7141-s003.docx]

Table S1

OTUs per functional group. Number of OTUs assigned to each functional group, compared to the total number of taxonomically annotated OTUs.

| Functional group | OTUs | Fraction(%) |
| --- | --- | --- |
| Chemoheterotrophy | 1425 | 28.15 |
| Aerobic chemoheterotrophy | 1319 | 26.05 |
| Predatory or exoparasitic | 215 | 4.25 |
| Nitrification | 169 | 3.34 |
| Ureolysis | 133 | 2.63 |
| Nitrate reduction | 104 | 2.05 |
| Animal parasites or symbionts | 98 | 1.94 |
| Nitrogen fixation | 94 | 1.86 |
| Human pathogens all | 93 | 1.84 |
| Aerobic ammonia oxidation | 90 | 1.78 |
| Phototrophy | 85 | 1.68 |
| Aerobic nitrite oxidation | 79 | 1.56 |
| Human pathogens pneumonia | 77 | 1.52 |
| Aromatic compound degradation | 74 | 1.46 |
| Nitrogen respiration | 74 | 1.46 |
| Photoautotrophy | 74 | 1.46 |
| Nitrate respiration | 72 | 1.42 |
| Cellulolysis | 58 | 1.15 |
| Respiration of sulfur compounds | 53 | 1.05 |
| Sulfur respiration | 52 | 1.03 |
| photoheterotrophy | 52 | 1.03 |
| Nitrite respiration | 45 | 0.89 |
| Nitrate denitrification | 43 | 0.85 |
| Nitrite denitrification | 43 | 0.85 |
| Nitrous oxide denitrification | 43 | 0.85 |
| Denitrification | 43 | 0.85 |
| Fermentation | 42 | 0.83 |
| Anoxygenic photoautotrophy S oxidizing | 41 | 0.81 |
| Anoxygenic photoautotrophy | 41 | 0.81 |
| Cyanobacteria | 33 | 0.65 |
| Oxygenic photoautotrophy | 33 | 0.65 |
| Intracellular parasites | 29 | 0.57 |
| Manganese oxidation | 27 | 0.53 |
| methylotrophy | 25 | 0.49 |
| Methanol oxidation | 15 | 0.30 |
| Chitinolysis | 15 | 0.30 |
| Hydrocarbon degradation | 12 | 0.24 |
| Methanotrophy | 10 | 0.20 |
| Dark oxidation of sulfur compounds | 5 | 0.10 |
| Iron respiration | 5 | 0.10 |
| Xylanolysis | 4 | 0.08 |
| Chloroplasts | 4 | 0.08 |
| Anammox | 2 | 0.04 |
| Dark hydrogen oxidation | 2 | 0.04 |
| Human gut | 2 | 0.04 |
| Mammal gut | 2 | 0.04 |
| Aromatic hydrocarbon degradation | 2 | 0.04 |
| Aliphatic non methane hydrocarbon degradation | 2 | 0.04 |
| Sulfate respiration | 1 | 0.02 |
| Dark sulfide oxidation | 1 | 0.02 |
| Dark thiosulfate oxidation | 1 | 0.02 |
