# Supplementary material for: Comparison of soil bacterial community and functional characteristics following afforestation in the semi-arid areas
Source: PeerJ. 2019 Jun 25;7:e7141. doi: 10.7717/peerj.7141 (PMC6598672; doi:10.7717/peerj.7141)
Supplement: Table S2 — Data are means ± standard error (n = 3). PC: Populus × canadensis Moench; PS: Pinus sylvestris var. mongolica; PT: Pinus tabuliformis. Different small letters meant significant difference at 0.05 level. Different big letters meant significant difference at 0.01 level. [file peerj-07-7141-s004.docx]

Table S2

Functional groups related to the carbon cycle.

| Samples | Chemoheterotrophy | | Aerobic chemoheterotrophy | | Phototrophy | | Photoautotrophy | | Photoheterotrophy | | Anoxygenic photoautotrophy S oxidizing | | Anoxygenic photoautotrophy |
| --- | --- | --- | --- | --- | --- | --- | --- | --- | --- | --- | --- | --- | --- |
| PC | 26.526±2.90aA | | 25.64±2.84aA | | 1.75±0.09aA | | 1.75±0.09aA | | 1.28±0.23aA | | 1.20±0.22aA | | 1.21±0.22aA |
| PT | 31.565±3.96aA | | 30.58±3.82aA | | 1.16±0.25bB | | 1.16±0.25bB | | 0.74±0.29bA | | 0.68±0.23bA | | 0.68±0.26bA |
| PS | 31.73±0.44aA | | 29.89±0.64aA | | 0.90±0.04bB | | 0.90±0.04bB | | 0.79±0.04bA | | 0.69±0.04bA | | 0.69±0.04bA |
| Samples | | Cellulolysis | | Oxygenic photoautotrophy | | Methylotrophy | | Methanol oxidation | | Hydrocarbon degradation | | Methanotrophy | |
| PC | | 0.89±0.58aA | | 0.47±0.14aA | | 0.20±0.10bA | | 0.17±0.07bB | | 0.04±0.03aA | | 0.04±0.03aA | |
| PT | | 0.67±0.19aA | | 0.42±0.08aA | | 0.21±0.10bA | | 0.13±0.04bB | | 0.08±0.07aA | | 0.08±0.07aA | |
| PS | | 0.38±0.12aA | | 0.11±0.02bB | | 0.38±0.03aA | | 0.33±0.04aA | | 0.09±0.02aA | | 0.05±0.02aA | |

Note:

Data are means ± standard error (n =3). PC: *Populus* × *canadensis* Moench; PS: *Pinus* *sylvestris* var. *mongolica*; PT: *Pinus* *tabuliformis*. Different small letters meant significant difference at 0.05 level. Different big letters meant significant difference at 0.01 level
