# Supplementary material for: Comparison of soil bacterial community and functional characteristics following afforestation in the semi-arid areas
Source: PeerJ. 2019 Jun 25;7:e7141. doi: 10.7717/peerj.7141 (PMC6598672; doi:10.7717/peerj.7141)
Supplement: Table S3 — Data are means ± standard error (n = 3). PC: Populus × canadensis Moench; PS: Pinus sylvestris var. mongolica; PT: Pinus tabuliformis. Different small letters meant significant difference at 0.05 level. Different big letters meant significant difference at 0.01 level. [file peerj-07-7141-s005.docx]

Table S3

Functional groups related to the nitrogen cycle.

| Samples | nitrification | | Nitrate reduction | Nitrogen respiration | Nitrate respiration | Aerobic nitrite oxidation | | Aerobic ammonia oxidation | |  |
| --- | --- | --- | --- | --- | --- | --- | --- | --- | --- | --- |
| PC | 5.39±1.23aA | | 3.81±0.24aA | 3.48±0.26aA | 3.47±0.25aA | 3.36±0.39aA | | 2.03±0.84aA | |  |
| PT | 4.60±1.37aA | | 3.04±0.67abAB | 2.82±0.71abA | 2.77±0.67abA | 2.73±0.63abA | | 1.87±0.827aA | |  |
| PS | 4.26±0.44aA | | 2.54±0.01bB | 2.23±0.08bA | 2.22±0.09bA | 2.29±0.173bA | | 1.96±0.32aA | |  |
| Samples | | Nitrogen fixation | Nitrite respiration | Nitrate denitrification | | Nitrite denitrification | Nitrous oxide denitrification | | denitrification | |
| PC | | 1.15±0.325aA | 1.18±0.23aA | 1.18±0.23aA | | 1.17±0.23aA | 1.17±0.23aA | | 1.18±0.23aA | |
| PT | | 1.185±0.495aA | 0.73±0.29bA | 0.68±0.27bA | | 0.68±0.27bA | 0.68±0.27bA | | 0.68±0.27bA | |
| PS | | 0.995±0.13aA | 0.72±0.03bA | 0.71±0.03bA | | 0.71±0.03bA | 0.71±0.03bA | | 0.71±0.03bA | |

Note:

Data are means ± standard error (n =3). PC: *Populus* × *canadensis* Moench; PS: *Pinus* *sylvestris* var. *mongolica*; PT: *Pinus* *tabuliformis*. Different small letters meant significant difference at 0.05 level. Different big letters meant significant difference at 0.01 level.
